# The influence of aging on the fracture load of milled monolithic crowns

**DOI:** 10.1186/s12903-022-02529-z

**Published:** 2022-11-19

**Authors:** Ceyda Güleç, Işıl Sarıkaya

**Affiliations:** Department of Prosthodontics, Faculty of Dentistry, Tokat Gaziosmanpasa University, Ali Şevki Erek Campus, 60100 Tokat, Turkey

**Keywords:** CAD/CAM, Chewing simulator, Aging, Fracture load, Monolithic crowns

## Abstract

**Background:**

This in-vitro study was conducted to assess the effect of aging on the fracture load of molar crowns fabricated with monolithic CAD/CAM materials.

**Methods:**

The crown restorations were produced from Cerasmart, Vita Enamic, and IPS e.max CAD blocks. Aging was applied to the 10 samples each of monolithic CAD/CAM materials (*n* = 10). Dual-axis chewing simulator (50 N, 1.1 Hz, lateral movement: 1 mm, mouth opening: 2 mm, 1,200,000 cycles) and thermocycling (± 5–55 °C, 6000 cycles) were applied as an aging procedure. 10 samples each of monolithic CAD/CAM materials without aging (*n* = 10) were considered the control group. 6 tested groups were obtained. Then, all samples were evaluated in a universal testing machine to determine the fracture loading values’.

**Results:**

There was not a statistically significant difference between the fracture load values before and after aging for all samples of Cerasmart, Vita Enamic, and IPS e.max CAD (*p* > 0.005). In a comparison of the monolithic materials together, a statistically significant difference was found between the fracture load values of IPS e.max CAD and Vita Enamic crowns before aging (*p* = 0.02). Also, Vita Enamic crowns (1978,71 ± 364,05 N) were found different from the IPS e.max CAD (*p* = 0.005) and Cerasmart crowns (*p* = 0.041) after aging.

**Conclusions:**

Dynamic aging with 1.200.000 cycles was found to have no effect to fracture loading on milled Cerasmart, Vita Enamic, and IPS e.max CAD monolithic crowns.

## Background

Increased interest and demand for biocompatible restorations that contain no metals have encouraged researchers to search for new materials. All of the ceramic restorations became promising with the help of soft tissue biocompatibility [1], improved color stability, improved abrasion resistance as well as superior light transmittance [2]. In recent years, computer-aided design (CAD) /computer-aided manufacturing (CAM) technologies have been extensive in dentistry. Standardized production processes of CAD/CAM-produced restorations have enabled uniform material quality, reproducibility of restorations, and reduced production costs. The elimination of the veneer layer by using monolithic materials makes a more conservative preparation possible on crown restorations. Another advantage of using monolithic ceramics is that significantly reduces the risk of cohesive failure, compared with conventional veneering techniques [3, 4].

Lithium disilicate glass–ceramics are usually used for monolithic restorations because of their high fracture resistance [5–7]. IPS e.max CAD-lithium disilicate blocks with high flexural strength of 360 MPa and modulus of elasticity of 95 GPa, first introduced in 2005 [8]. The presinterised block contains 40% of lithium meta-silicate crystals about 0.5 µm-crystalline sizes. Depending on the amount of colorant, the ceramic is blue-colored in this phase with its 130–150 MPa-flexural strength. After this blue block is processed as a dental restoration, lithium meta-silicate is turned into lithium disilicate is done at 850 °C for 25 min by the crystallization process. Glass–ceramic contains 70% lithium disilicate by volume at this stage.

Recently, CAD-CAM blocks of interpenetrating ceramic and polymer networks (PICN) have been introduced [9–11]. This dual-network provides reduced brittleness and surface hardness, and elastic modulus closer to that of dentin, faster milling, better marginal quality, and easier polishing [6, 11]. Vita Enamic is one of the PICN materials with contains a sintered-glass ceramic network of 86% by weight, and by 75% by volume. With its 30 GPa elasticity, it is very close to natural dentin [12]. Cerasmart, another nanoceramic CAD/CAM blocks (20 µm-silica, and 300 nm-barium glass) contain fillers of 71% by volume and has a flexural strength of 231 MPa and bending strength of 7.5 GPa [13]. There is no need for a glazing or sintering process for Cerasmart crowns. Also mechanically, it is considered to be from the same family as PICN materials. However, inadequate data exist regarding the fatigue resistance of posterior hybrid ceramic restorations [9, 10, 14–16].

The primary criterion in determining the restorative material used is that it has sufficient mechanical properties against chewing forces and can protect the remaining tooth structure. To determine the durability of all-ceramic restorations similar to the oral conditions, loading them under similar thermal and mechanical conditions is beneficial. Restorations are loaded repeatedly in the presence of water with masticatory loads reaching an average of 240.000–250.000 cycles/year [17–22]. However, there is little information on the long-lasting mechanical load properties of monolithic CAD/CAM polymer-based resin composite materials [23, 24]. In a study, resin-based CAD/CAM materials were found to have higher fatigue resistance to occlusal loads than CAD/CAM ceramic materials [25]. In addition to fatigue loading, artificial aging with a thermal cycle is a well-established method to imitate the clinical situation [26]. There are many problems with restoration due to temperature changes in the oral cavity caused by eating, drinking, and breathing [2]. Different thermal cycle temperatures were used and most of these temperatures range from + 5 °C to + 55 °C in the in vitro studies [27–29]. In addition, various cycles ranging from 1 to 1,000,000 and retention times ranging from 4 s to 20 min have been observed [30]. Also, there is a need for long-term work on fatigue behavior, and fracture resistance of new hybrid ceramic materials.

This in-vitro study was conducted to assess the fracture load of monolithic CAD/CAM molar crown restorations after aging. The null hypothesis of the study was that the fracture load values would reveal no significant difference before and after aging on milled crown restorations fabricated with the lithium disilicate and hybrid monolithic CAD/CAM ceramics.

## Methods

### Preparation of specimens

Sixty freshly extracted and caries-free left mandibular first human molar teeth were collected, cleaned, and stored in 0.1% thymol solution [31]. The dimensions of the collected teeth were measured as11 ± 1 mm in the mesiodistal direction, 10 ± 1 mm in the labio-lingual direction, and 7 ± 1 mm in the cervical-occlusal direction. Then, the teeth were prepared according to the accepted tooth preparation principles using a chamfer diamond rotary instrument (879 014 10: Diatech Dental AG:Heerbrugg:Switzerland) by adjusting for a 1 mm circumferential chamfer margin, 1.5 mm occlusal reduction, 1 mm axial preparation, and 6° convergence angle [32]. After preparation, the master casts were evaluated using a surveyor to detect undercuts.

Subsequently, the teeth were divided into three test groups randomly (*n* = 20). For each test group, full-crown restorations were fabricated with A2 HT- Cerasmart (Cerasmart: GC: Tokyo: Japan), 2M2 HT- Vita Enamic (Vita Enamic: Vita Zahnfabrik: Bad Sackingen: Germany), and A2- IPS e.max CAD (IPS e.max CAD: Ivoclar Vivadent: Liechtenstein) monolithic blocks in Cerec System (Cerec System: Sirona: Bensheim: Germany) by the same laboratory technician. Prepared teeth were mounted to an optic reader in the Cerec System after application of reflectance opaque powder (Cerec Optispray: Sirona: Bensheim: Germany). Design of full crowns of lithium disilicate and hybrid ceramics was made onto the scanned models. Tooth number- 36, crown type, and design were selected from the gallery for fabrication of full crown restoration. The finish line of the restoration was determined. 2-mm occlusal reduction was set on crests of cusps, 1.5-mm on the central fossa, and 1 mm on the cervical finish line to simulate the original tooth morphology of number- 36. Data was sent to the Cerec InLab milling machine (Cerec InLab MCXL: Sirona: Bensheim: Germany) for the fabrication of full crowns. Sintering and glazing were applied to IPS e.max CAD crowns at 850 °C in an inLab furnace (Cerec inLab Profire: Sirona: Bensheim: Germany) for 25 min. Cerasmart and Vita Enamic specimens were first sandblasted with 25–50 µm Al_2_O_3_, and then Ceramic Primer II (GC: Tokyo: Japan) was applied, then air-dried. After that Optiglaze Color (Optiglaze Color: GC: Tokyo: Japan) was applied to the specimens, and polymerized with LED (Elipar Deepcure-S: 3 M Espe: St. Paul: USA) curing.

### Luting of the crowns

All the crown restorations were adhesively luted on prepared molar teeth using a dual-cure composite material (Panavia F 2.0: Kuraray Noritake Dental Inc: Tokyo: Japan). Equal amounts of Panavia Paste A and B (Panavia F 2.0: Kuraray Noritake Dental Inc: Tokyo: Japan) were mixed and applied to the inner surfaces of the crowns according to the manufacturer’s instructions. The restorations were seated onto the teeth and held in place by the application of the same operator’s finger pressure [33–36]. The excess cement was removed with sponge pellets, and an air-blocking gel (Oxiguard II: Kuraray Noritake Dental Inc: Tokyo: Japan) was applied. Then they were cured (Elipar Deepcure-S: 3 M Espe: St. Paul: USA) for 20 s. The specimens were stored for 24 h at 37 °C before being subjected to aging.

### Aging

All the root surfaces of the teeth were coated with a 1 mm-thick polyether layer (Impregum Soft: 3 M Espe: Seefeld: Germany) from the marginal finish line of the restorations to 2-mm apical direction to simulate the physiologic mobility of teeth [37, 38]. The teeth were immersed in a wax bath, which was replaced by polyether in a second fabrication process, as previously described. Later, restorations on teeth were fixed in a resin mold, which acts as the sample holder for the chewing simulator, using a self-curing acrylic resin material (Meliodent: Heraeus Kulzer: Hanau: Germany). Thermal and aging were not applied to half of the specimens (*n* = 10). The other half of the specimens underwent thermocycling (SD Mechatronik Thermocycler: SD Mechatronik GmbH: München: Germany) for 6,000 cycles between 5° and 55 °C, over a dwell time of 60 s, and a transfer time of 10 s (*n* = 10) [39, 40]. After thermocycling, the specimens were subjected to a 2-body wear test in a dual-axis chewing simulator in distilled water solution (CS 4.2: SD Mechatronic GmbH: München: Germany). Steatite balls (Hoechst Ceram Tec.: Wunsiedel: Germany) of 6 mm diameter were used as the opposing occlusal surface. The balls were fixed to the upper sample holders of the chewing simulator using a light-curing composite resin (GC Pattern Resin: GC: Tokyo: Japan). The chewing simulation parameters used are summarized in Table 1. The load was transferred to the center of the central fossa of the mandibular first crowns by antagonistic steatite balls. To simulate 5-years of clinical service, a total of 1,200,000 cycles were performed [27, 28, 41].

**Table 1:** Configuration of parameters set for aging

Parameter	Data
Number of cycles	1.200.000
Force	49 N
Height	2 mm
Lateral movement	1 mm
Descendent speed	55 mm/s
Lifting speed	55 mm/s
Feed speed	50 mm/s
Return speed	50 mm/s
Frequency	1.1 Hz

### Fracture load test

Following the aging procedure, the specimens were tested on a universal testing machine (AGS-X: Shimadzu: Tokyo: Japan) until fracture. They were subjected to a compressive force at a crosshead speed of 0,5 mm/min with a round-shaped modified bur of 4 mm diameter. A metal bar was positioned parallel to the long axes of the crown specimens and the buccal and lingual cusps of the crowns were used to apply the force. The maximum load necessary to fracture each specimen was recorded in newtons (N).

### SEM

To characterize the surface wear patterns, one specimen of each monolithic CAD-CAM crown group was evaluated by a scanning electron microscopy (SEM-JEOL JSM–7001F: Jeol Ltd.: Tokyo: Japan) after the fracture loading test, for which the sample surfaces were initially coated (Quorum SC7620: Quorum Tech Ltd.: Newhaven: UK) with a thin layer of 18 kt-gold (15.9 g / ml). The surfaces were then examined at a magnification of 10 × at 25 keV.

### Statistical analysis

Statistical analysis was performed using SPSS 20.0 (IBM SPSS Statistics 20: IBM Co: Somers: NY: USA) for Windows. Having assessed that, all the obtained results were normally distributed and the differences in the measures in terms of groups were evaluated using repeated measures of Variance analysis. The results are expressed as mean ± standard deviation and the level of significance is set at 5% (*p* < 0.05).

## Results

None of the samples fractured during aging. A two-way ANOVA test was used for the comparison between fracture values of each material before and after aging within itself groups of Cerasmart, Vita Enamic, and IPS e.max CAD. The mean and standard deviation of the Fracture Load values are shown in Table 2. Mean values and standard deviations (SD) for Fracture Load of the Ceramart crowns before aging was 2731.81 ± 488.51 N, and after aging was 2578.99 ± 575.9 N, and there was no statistically significant difference compared to between of them (*p* > 0.05). Mean values and SD for Fracture Load of the Vita Enamic crowns before aging was 2195.46 ± 387.83 N, and after aging was 1978.71 ± 364.05 N, and there was no statistically significant difference compared between them (*p* > 0.05). Mean values and SD for Fracture Load of the IPS e.max CAD crowns before aging was 3098.4 ± 667.09 N, and after aging was 2781.51 ± 559.45 N, and there was no statistically significant difference compared to between them (*p* > 0.05).

**Table 2:** Mean values and standard deviations (SD) for Fracture Load (N) of the specimens

Material	Without aging	With aging
IPS e.max CAD	3098.4 ± 667.09 (a,x)	2781.51 ± 559.45 (a,x)
Cerasmart	2731.81 ± 488.51 (ab,x)	2578.99 ± 575.9 (a,x)
Vita Enamic	2195.46 ± 387.83 (b,x)	1978.71 ± 364.05 (b,x)

^*^Different letters indicate a statistically significant difference between groups (*p* < 0.05)

^*^a,b intra-group comparisons

^**^x,y between-group comparisons

Comparison of the monolithic materials together with the two-way ANOVA showed statistically significant differences regarding the fracture load values of IPS e.max CAD, Cerasmart, and Vita Enamic crowns after 1,200,000 chewing cycles were analyzed (*p* < 0.05). While the load value for the highest fracture was observed in IPS e.max CAD ( 3098,4 ± 667,09 N) crowns that aging was not applied, the lowest fracture load value was observed in aging applied-Vita Enamic ( 1978,71 ± 364,05 N) crowns. Multiple comparisons for Fracture Load values of specimens that aging was not applied are shown in Table 3. There was not a statistically significant difference between the fracture load values of specimens of IPS e.max CAD and Cerasmart crowns that aging was not applied (*p* = 0.395). Likewise, there was not a statistically significant difference between the fracture load values of specimens of Cerasmart and Vita Enamic crowns to which aging was not applied (*p* = 0.093). However, it was found that there existed a statistically significant difference between the fracture load values of specimens of IPS e.max CAD and Vita Enamic crowns to which aging was not applied (*p* = 0.02). Multiple comparisons for Fracture Load values of specimens that aging was applied are shown in Table 4. According to the fracture load values after the aging process, there was not a statistically significant difference between the fracture load values of IPS e.max CAD crowns ( 2781,51 ± 559,45 N) and Cerasmart (2578,99 ± 575,9 N) crowns to which aging was applied (*p* = 1). The fracture load values of Vita Enamic crowns (1978,71 ± 364,05 N) were statistically significantly different from both the fracture load values of IPS e.max CAD (*p* = 0.005) and Cerasmart crowns (*p* = 0.041) to which aging was applied.

**Table 3:** Multiple comparisons for Fracture Load values of specimens that aging was not applied

	**(I) Material**	**(J) Material**	**Average difference** **(I-J)**	**SD**	p^**^	**95% confidence interval for the difference**	
						**Lower limit**	**Upper limit**
**Aging was not applied**	IPS e.max	Cerasmart	366.590	235.806	.395	-235.296	968.476
		Enamic	902.941^a^		.002	301.055	1504.827
	Cerasmart	IPS e.max	-366.590		.395	-968.476	235.296
		Enamic	536.351		.093	-65.535	1138.237
	Enamic	IPS e.max	-902.941^a^		.002	-1504.827	-301.055
		Cerasmart	-536.351		.093	-1138.237	65.535

^a^0.05 level of significance

^**^Bonferroni correction for multiple comparison

**Table 4:** Multiple comparisons for Fracture Load values of specimens that aging was applied

	**(I) Material**	**(J) Material**	**Average difference (I-J)**	**SD**	p^**^	**95% confidence interval for the difference**	
						**Lower limit**	**Upper limit**
**Aging was applied**	IPS e.max	Cerasmart	202.519	227.624	1.000	-378.481	783.519
		Enamic	802.798^a^		.005	221.798	1383.798
	Cerasmart	IPS e.max	-202.519		1.000	-783.519	378.481
		Enamic	600.279^a^		.041	19.279	1181.279
	Enamic	IPS e.max	-802.798^a^		.005	-1383.798	-221.798
		Cerasmart	-600.279^a^		.041	-1181.279	-19.279

^a^0.05 level of significance

^**^Bonferroni correction for multiple comparisons

**Fig. 1:**
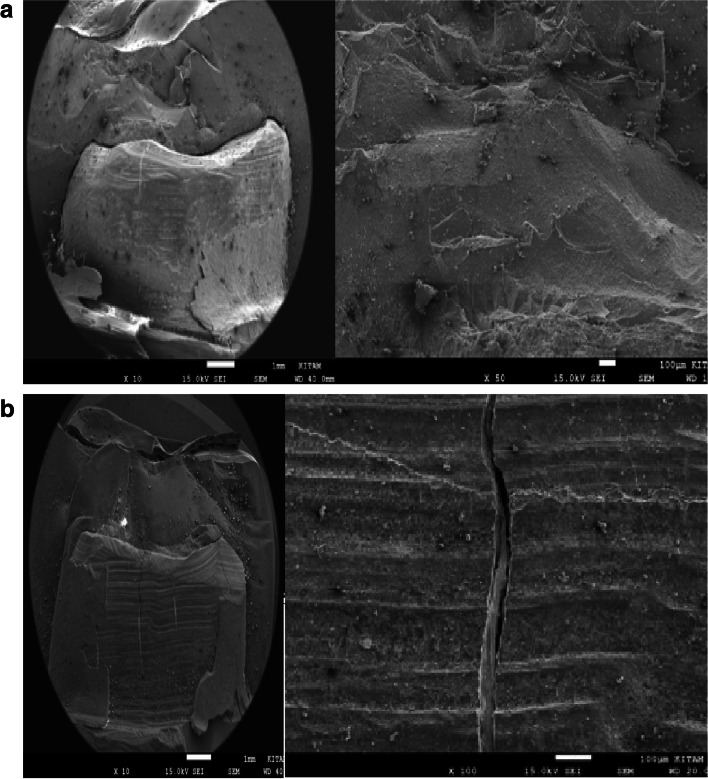
**a** SEM images of the non-aged IPS e.max CAD specimen’s. **b** SEM images of the aged IPS e.max CAD specimen’s

**Fig. 2:**
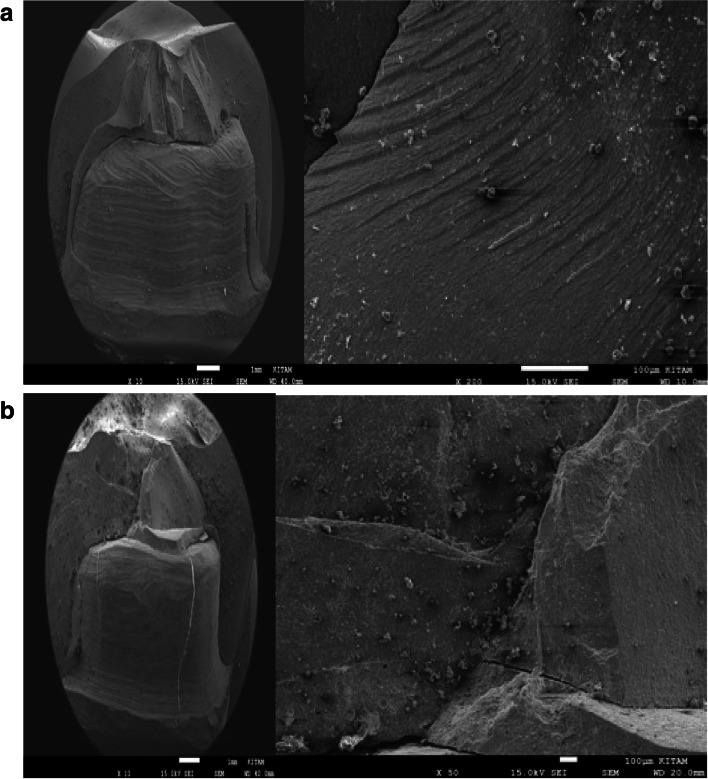
**a** SEM images of the non-aged Cerasmart specimen’s. **b** SEM images of the aged Cerasmart specimen’s

**Fig. 3:**
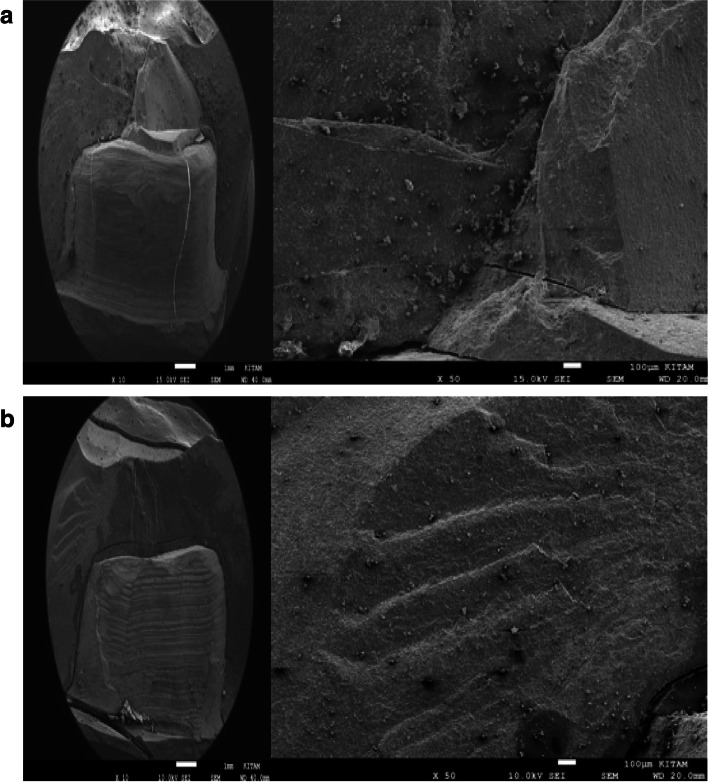
**a** SEM images of the non-aged Vita Enamic specimen’s. **b** SEM images of the aged Vita Enamic specimen’s

Representative SEM images of × 10 magnifications of the surface patterns of Cerasmart, Vita Enamic, and IPS e.max CAD crowns’ before and after aging are shown in Figs. 1, 2 and 3 (a,b).

## Discussion

To overcome the chipping behavior of the newly developed esthetic ceramics, the use of monolithic materials has become widespread. In this study, the fracture load of monolithic molar crown restorations fabricated with lithium disilicate reinforced glass–ceramic and hybrid ceramic blocks after aging was investigated in vitro conditions. In the light of the results of this study, although a decrease was observed in the mean fracture load values of all samples with aging compared to those without aging, this decrease was not significant for all materials. For this reason, the null hypothesis of the study—the fracture load values would reveal no significant difference before and after aging on milled crown restorations fabricated with the lithium disilicate and hybrid monolithic CAD/CAM ceramics—was accepted. In the present study, thermal cycling applications were performed with water temperatures of 5 °C and 55 °C over a dwell time of 60 s, a transfer time of 10 s, and a cycle number of 6000 cycles [39]. According to the literature, an average of 250,000 chewing cycles in a chewing simulator corresponds to use in a one-year clinical setting [42]. For this reason, 1,200,000 cycles were conducted in this study for the chewing function to correspond to five years [20, 21]. The chewing force 50 N at a frequency of 1–1.6 Hz has been commonly applied to simulate intraoral conditions in in-vitro studies [21, 22, 26, 32, 37].

Güngör and Nemli [22] have investigated the effect of aging on fracture resistance of monolithic ceramics and veneered zirconia crowns. The Vita Enamic crowns were catastrophically broken during the aging process. The highest fracture resistance values were found in the monolithic zirconia crowns, followed by IPS e.max CAD crowns, which were monolithic lithium disilicate. In this study, it is important to note that the application of aging with a force of 100 N, and the only polishing application instead of glazing on the specimens exhibit the fracture load values of tested ceramic blocks. This study is the first one advocating the use of resin ceramics as a veneer on the core. Contrary to this work, aging and deterioration can occur without visible catastrophic failures. In these cases, the next static fracture test can help detect weak spots. For this purpose, no fracture was observed in the present study during the dynamic loading with the chewing simulator. All surviving samples were placed in a universal test machine for static loading so that the fracture load values could be determined. Fracture load data cannot be directly related to clinical survival but may provide information on the suitability of new ceramics for the requirements of clinically proven systems.

In another study, the fracture resistances of six different restoration materials produced by CAD/CAM were compared [7]. Cerasmart, Lava Ultimate, and Paradigm MZ100 were found to be significantly more successful than the new hybrid blocks. Ceramic materials are less flexible and more fragile than blocks containing resin. This difference in the elastic property is caused by the resin component which helps to reduce the fragility. Materials that perform well in flexural testing should be investigated for other properties, such as cyclic fatigue, color stability, and material and antagonist wear. Yet, the materials which were used in these tests do not show the clinical setting [43]. For this reason, it is aimed to compare the effect of aging processes applied with using full-crown restorations on fracture load in the present study.

Aboushelib et al. [11], investigated the effect of cyclic fatigue on resin infiltrated ceramics and reinforced glass–ceramic blocks and reported that dynamic fatigue significantly reduced initial fracture strength. Among the resin-infiltrated ceramics, Lava Ultimate and Vita Enamic were less affected by fatigue and fracture strength while the incidence of fracture during fatigue was highest in resin-infiltrated ceramics. According to the results of the present study, it was found that there was not a significant difference between the mean fracture load values of the samples before and after aging. However, the reduction in the fracture strength of the lithium disilicate-reinforced ceramic samples was observed more than in the resin-containing ceramics similar to the study of Aboushelib et al. [11].

In the present study, fracture load revealed for all the crown retorations have a consistent crack pattern. Although there was no chipping, the milled monolithic crowns were massively (catastrophically) broken up to the surface of the prepared tooth. One limitation of this study is that the ceramic material does not have a uniform thickness. One of the reasons for the non-uniform thickness of the crown is the production of anatomically contoured crowns on standard preparations. Another limiting factor of this study may be the use of steatite antagonists instead of human tooth antagonists for dynamic loading. Further studies may be done for the fracture load of monolithic crowns opposing the human tooth antagonist with more cycles on the chewing simulator.

## Conclusions

In the light of the data obtained from the study that contributed to the literature by aging milled monolithic crowns in the posterior region, it was observed a decrease in the mean fracture load values of all samples with aging compared to those without aging, this decrease was not significant for all tested hybrid ceramic and lithium disilicate ceramic materials. None of the samples fractured during the aging of 1,200,000 cycles chewing simulator to simulate 5 years of clinical service. But the long-term mechanical behaviors of these monolithic CAD/CAM materials should be confirmed by future in-vitro and in-vivo investigations.

## Data Availability

The datasets generated and/or analyzed during the current study are not publicly available due to “The study was carried out as a specialization thesis in the field of prosthodontics” but are available from the corresponding author on reasonable request”.

## Abbreviations

CAD: Computer-aided design
CAM: Computer-aided manufacturing
%: Percent
°: Degree
°C: Centigrade degrees
h: Hour
min: Minute
sec: Second
mm: Millimeter
µm: Micrometer
N: Newton
MPa: Megapascal
GPa: Gigapascal
keV: Kiloelectron volt
kt: Karat
Hz: Hertz
Al_2_O_3_: Aluminum
PICN: Polymer infiltrated the ceramic network
